# Structural and compositional data of maya pottery samples from Lake Petén Itzá, Guatemala: Central America

**DOI:** 10.1016/j.dib.2021.106886

**Published:** 2021-02-16

**Authors:** Kefa K. Onchoke, Pressley S. Nicholson, Josephine Taylor, Robert R. Friedfeld, Leslie G. Cecil

**Affiliations:** a Department of Chemistry & Biochemistry, Stephen F. Austin State University, Box 13006 – SFA Station, Nacogdoches, TX, 75962-13006, USA; b Department of Biology, Stephen F. Austin State University, Box 13003 SFASU, Nacogdoches, TX 75962, USA; c Department of Physics, Engineering and Astronomy, Stephen F. Austin State University, Nacogdoches, TX, 75962, USA; d Department of Anthropology, Geography, and Sociology Stephen F. Austin State University, Box 13047 – SFA Station, Nacogdoches, Texas, 75962, USA

**Keywords:** Lake Petén Itzá, Guatemala, Pottery sherds, FTIR, SEM/EDX, XRD, TGA

## Abstract

In this data article, we present the spectroscopic structural data of the pottery samples collected from Petén Itzá, Guatemala. Detailed Fourier transmission infrared, powder X-ray diffraction, scanning electron microscopy coupled to electron dispersive X-ray diffraction, and thermal gravimetric analysis/differential thermal gravimetric analysis (FTIR, PXRD, SEM/EDX, and TGA/DTGA) were discussed in the research article titled “Comprehensive Structural and Compositional Investigation of Maya Pottery Sherds from Lake Petén Itzá, Guatemala, Central America” (Onchoke et al. 2020 [1])*.* The FTIR, XRD profiles and composition of pottery from Petén Itzá, Guatemala are presented. This data is important and useful for further understanding of the structural composition of pottery sherds used by Maya people of Guatemala. In addition, the TGA/DTGA profiles provide information on the content of the losses upon heating and offers supportive evidence to the spectroscopic data studied.

## Specifications Table

**Table:** 

Subject	*Analytical Chemistry*
Specific subject area	*Analytical Archaeology*
Type of data	*Table, graph, figure*
How data was acquired	(a) Fourier Transform Infrared (FTIR, Perkin Elmer Station 100 Inc., USA) was used for infrared analysis. (b) A Bruker AXS D8 Advance diffractometer equipped with an X-ray tube (Cu K_α_ radiation: λ = 1.54060 Å, 40 kV, and 40 mA) using a Ni filter and one-dimensional LynxEye detector at scanning speed of 2 °/min and 0.0125 ° step sizes and a 1s/step. (c) A JEOL-JSM 6100 scanning electron microscope equipped with a Horiba scanning electron microscopy/energy dispersive X-ray spectroscopy (SEM/EDX) was used. (d) A Perkin Elmer thermogravimetric and differential thermogravimetric analysis (TGA/DTGA) were performed with thermogravimetric simultaneous thermal analyzer (STA 6000) at 20°C/min heating rates in a nitrogen atmosphere in the range 34°C - 1000°C.
Data format	Raw data, analyzed
Parameters for data collection	(a) Power X-ray Diffraction (PXRD) analysis: Pottery samples obtained from Lake Petén Itzá, Guatemala were crushed, air dried, and ground to fine powder. (b) Fourier Transform infrared (FTIR) Analysis: The Diffuse Reflectance Infrared Fourier Transform Spectroscopy (DRIFTS) spectra in the 230 - 4000 cm^−1^ region was acquired on an abrasive pad (4 cm^−1^ resolution) with Perkin Elmer Spectrum 100 spectrometer equipped with a Ge/CsI beam splitter and a DTGS detector. (c) SEM/EDX analysis: SEM micrographs and their elemental composition were acquired with use of a JEOL-JSM 6100 scanning electron microscope.
Description of data collection	Forty-two pottery sherds were spectroscopically characterized and their crystalline phases determined. The powder diffraction file was acquired using Bruker AXS DIFFRAC.EVA program [2]. The fitted line profiles, peak search methods, and indexing of the lines were used to calculate the mineral identification via comparisons with the diffraction patterns with TOPAS program [3].
Data source location	Stephen F. Austin State University
Data accessibility	All data are available within this article.
Related research article	“Comprehensive Structural and Compositional Investigation of Maya Pottery Sherds from Lake Petén Itzá, Guatemala, Central America”, Kefa K. Onchoke, Pressley S Nicholson, Leslie G. Cecil, Robert B. Friedfeld, Josephine Taylor, Paul W. Weatherford [1].

## Value of the Data

• The spectral data provided here is valuable for referencing, identification of crystalline phases, and differentiation between the pottery samples from sites around the world.
• The data provides important information for identification of elemental compositions in pottery sherds and samples.
• The powder X-ray diffraction (PXRD) patterns are important for the identification of crystalline phases in pottery, and for comparisons to other archaeological samples.

## 1 Data Description

**Fig. 1:**
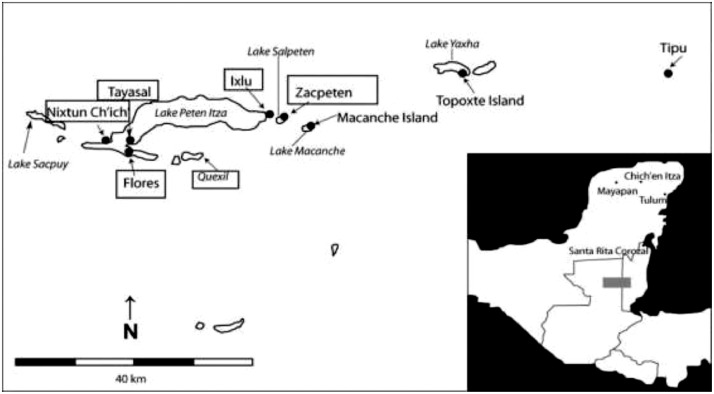
Map of Guatemala; Picture adopted from Ref # [4]. Reprinted with permission from Elsevier publisher. Elsevier License for permission to re-use in a journal article was granted from the Elsevier publisher.

**Fig. 2:**
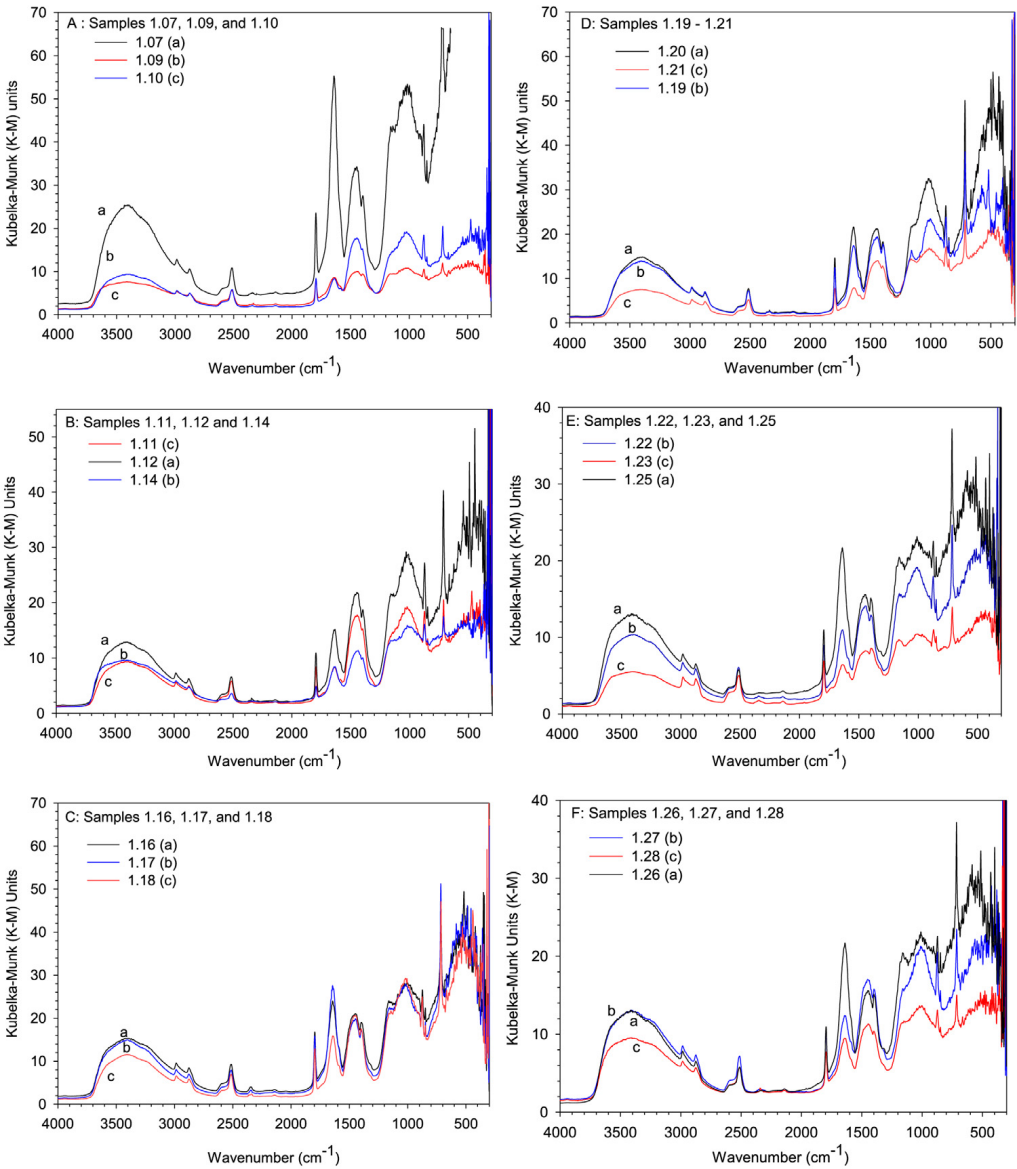

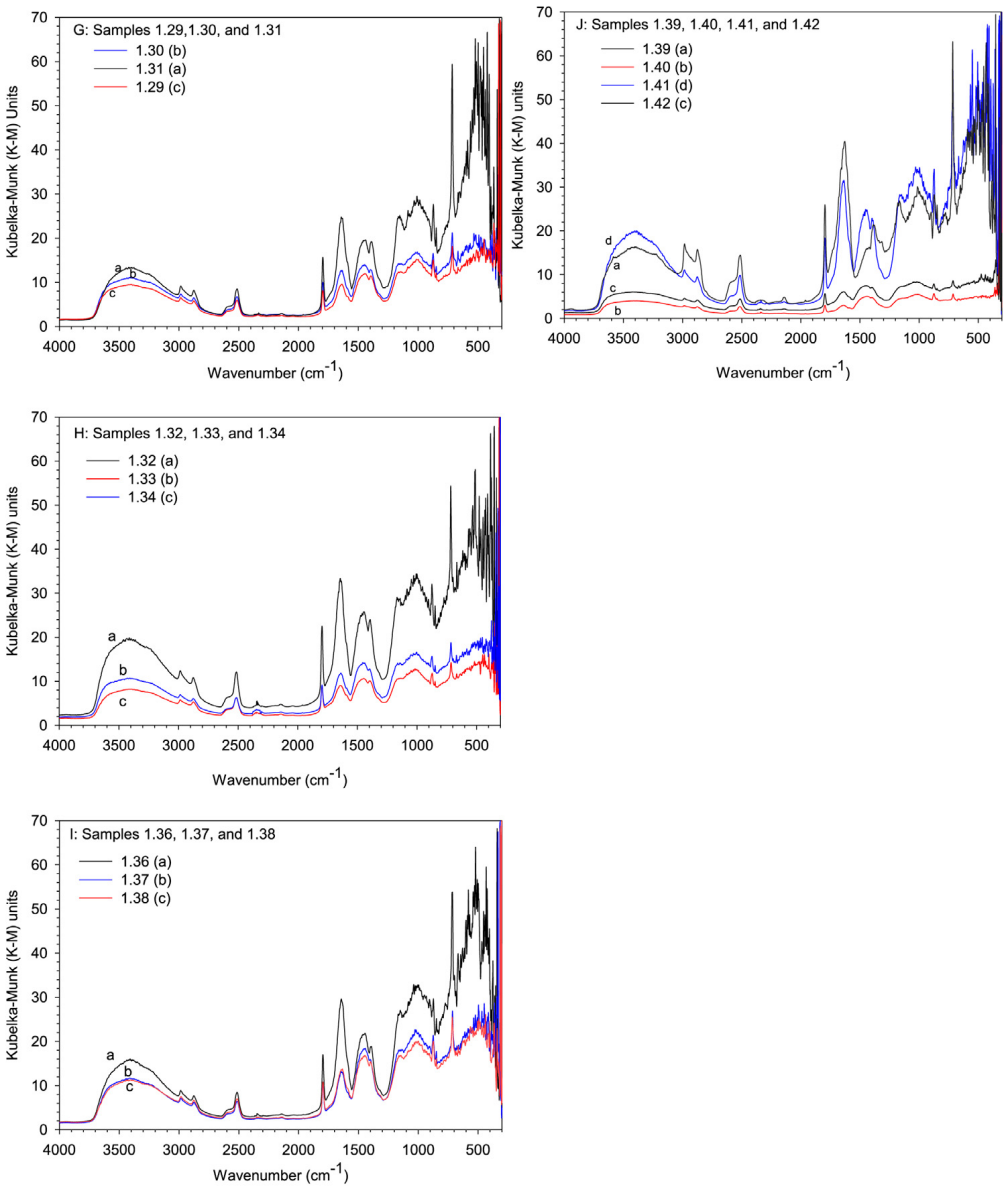
The FTIR data for pottery samples numbered (A) 1.07, 1.09, 1.10, (B) 1.11, 1.12, 1.14, (C) 1.16 -18, (D) 1.19 -1.21, (E) 1.22, 1.23, 1.25, (F) 1.26 -1.28, (G) 1.29 -1.31, (H) 1.32 – 1.34, (I) 1.36 -1.38, and (J) 1.39 -1.42. The traces are overlain to show their spectral similarities.

**Fig. 3:**
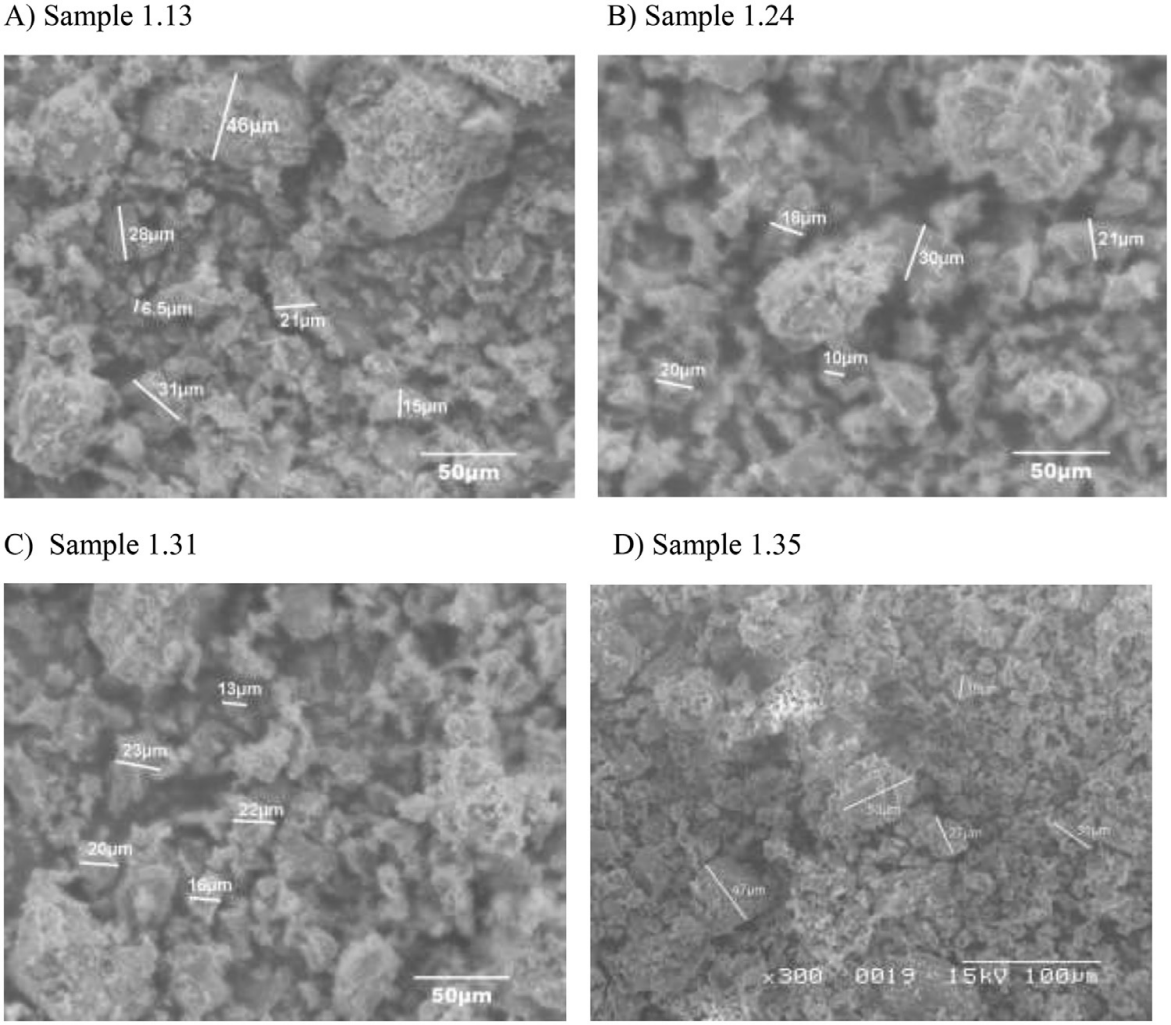
Representative SEM micrograph of pottery samples (numbered 1.13 (A), 1.24 (B), 1.24 (C), and 1.35 (D)) from Guatemala showing particle size diameters at magnification 400X and 300X, Voltage applied =15 kV, 20 kV.

**Fig. 4:**
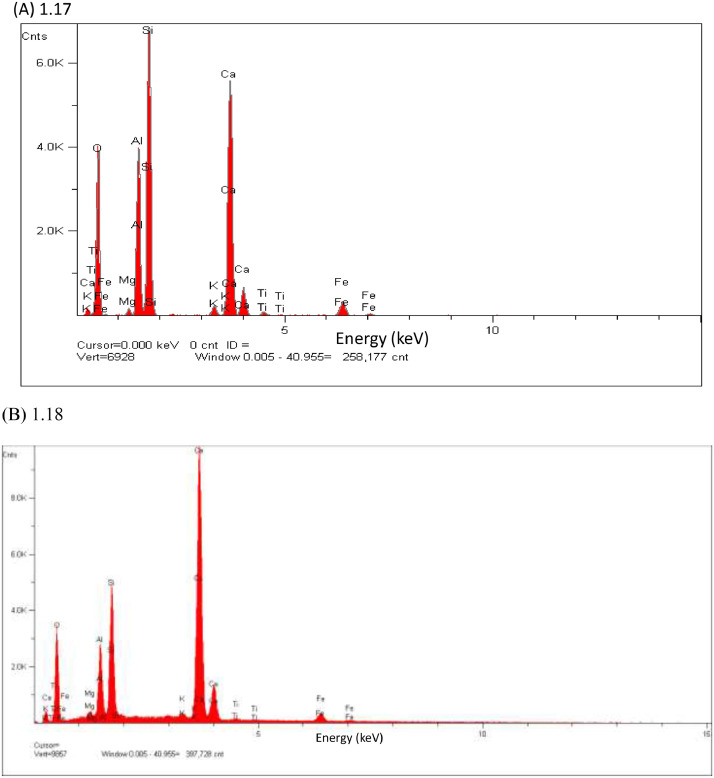

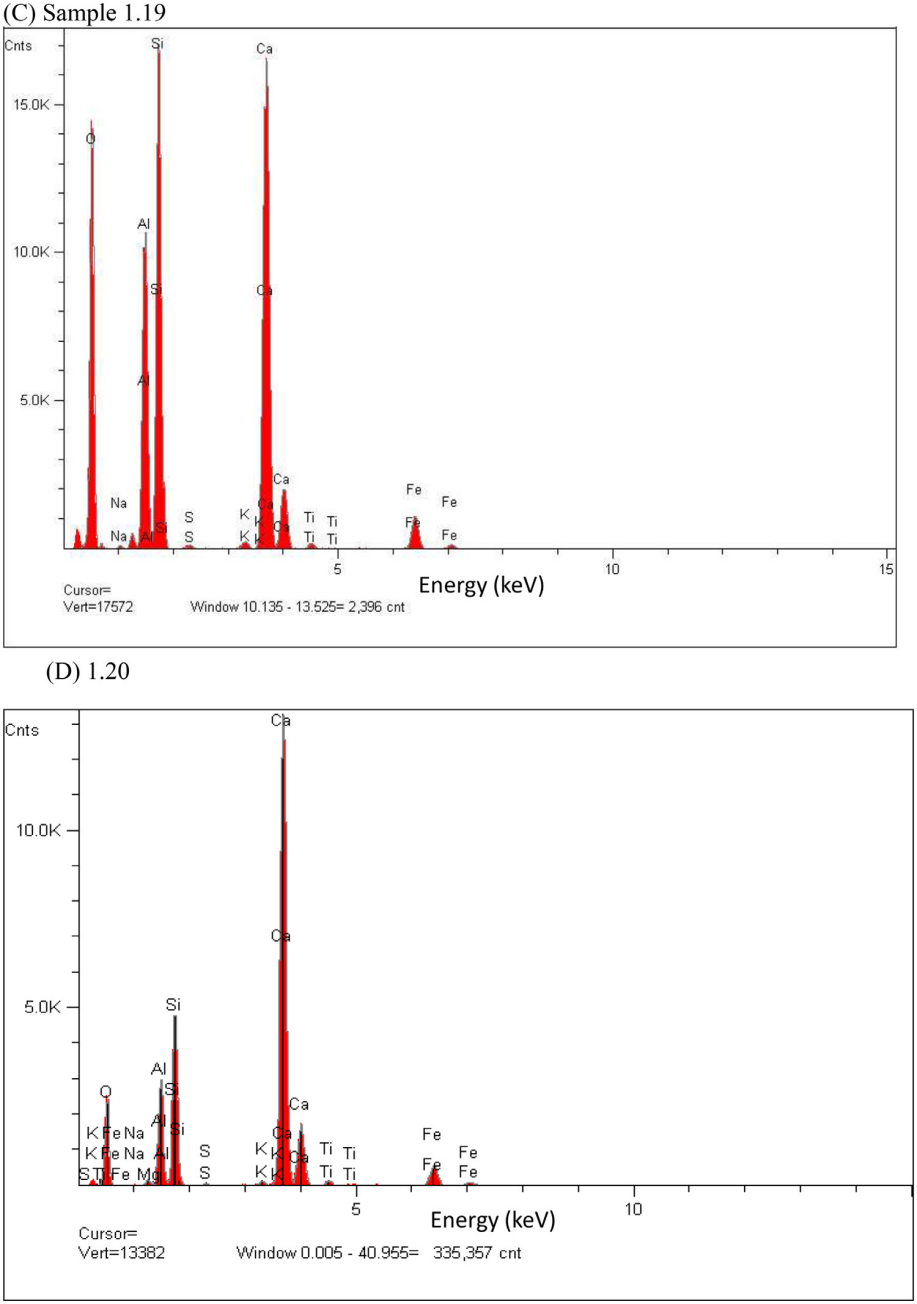

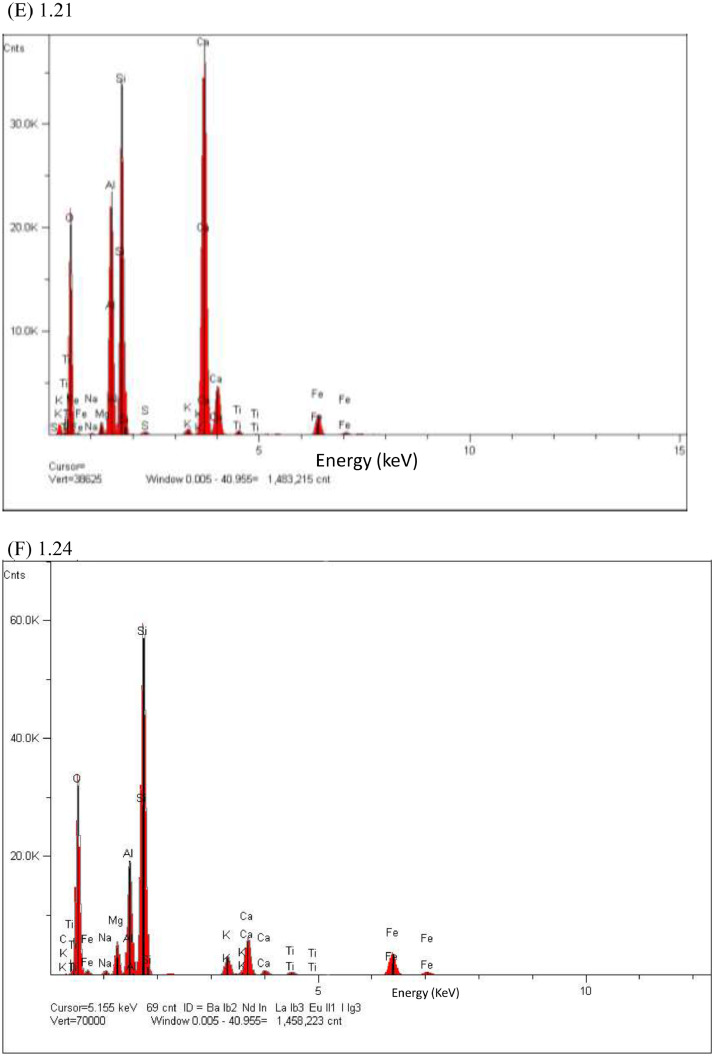

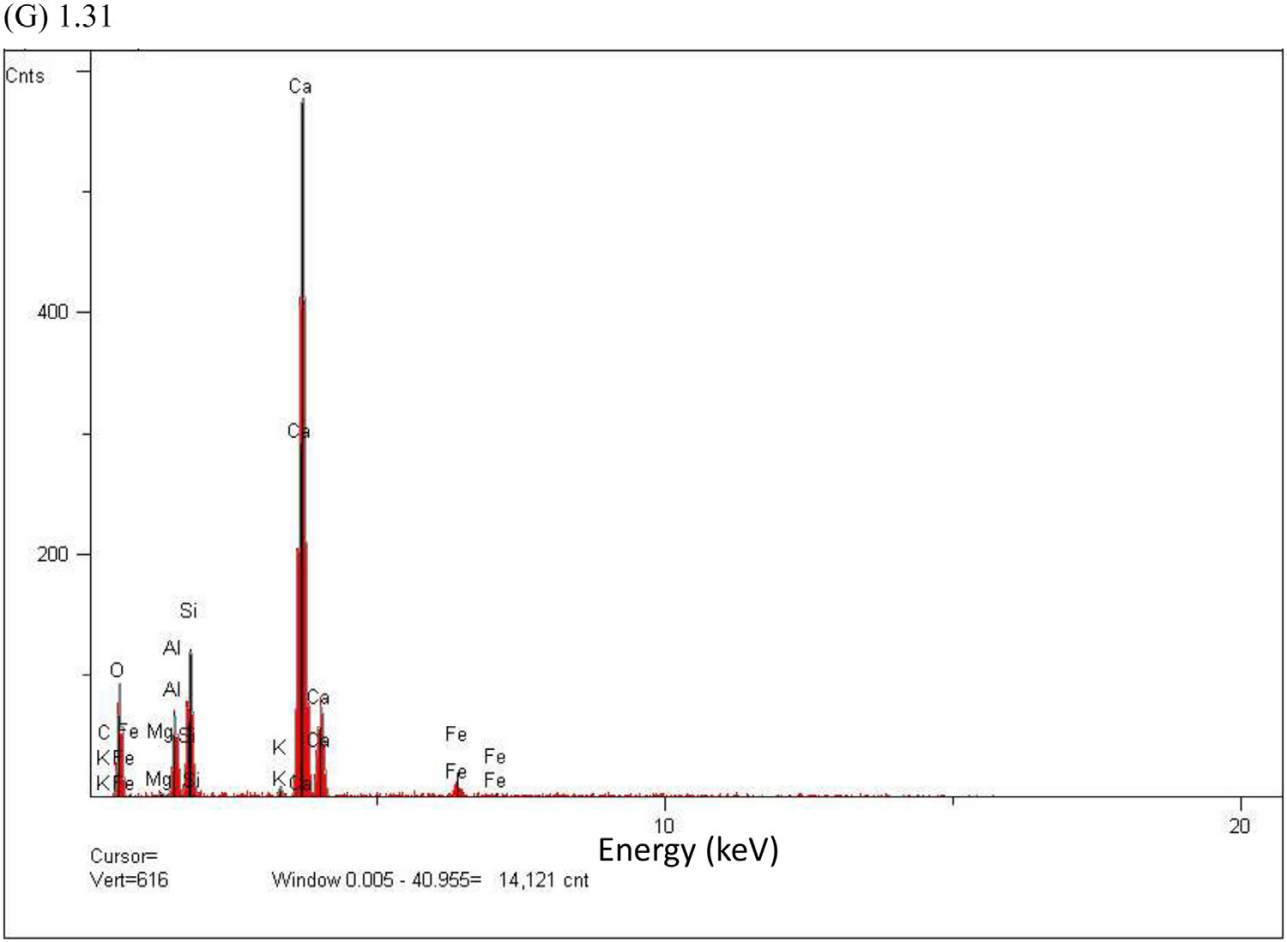
EDX elemental analysis spectra of pottery sample number 1.17 (A), 1.18(B), 1.19 (C), 1.20 (D), 1.21(E), 1.24 (F), 1.31 (G). **Fig. 4 (Cont'd**): EDX elemental analysis spectra of pottery sample number 1.19 (C), 1.20 (D) are continued to next page.

**Fig. 5:**
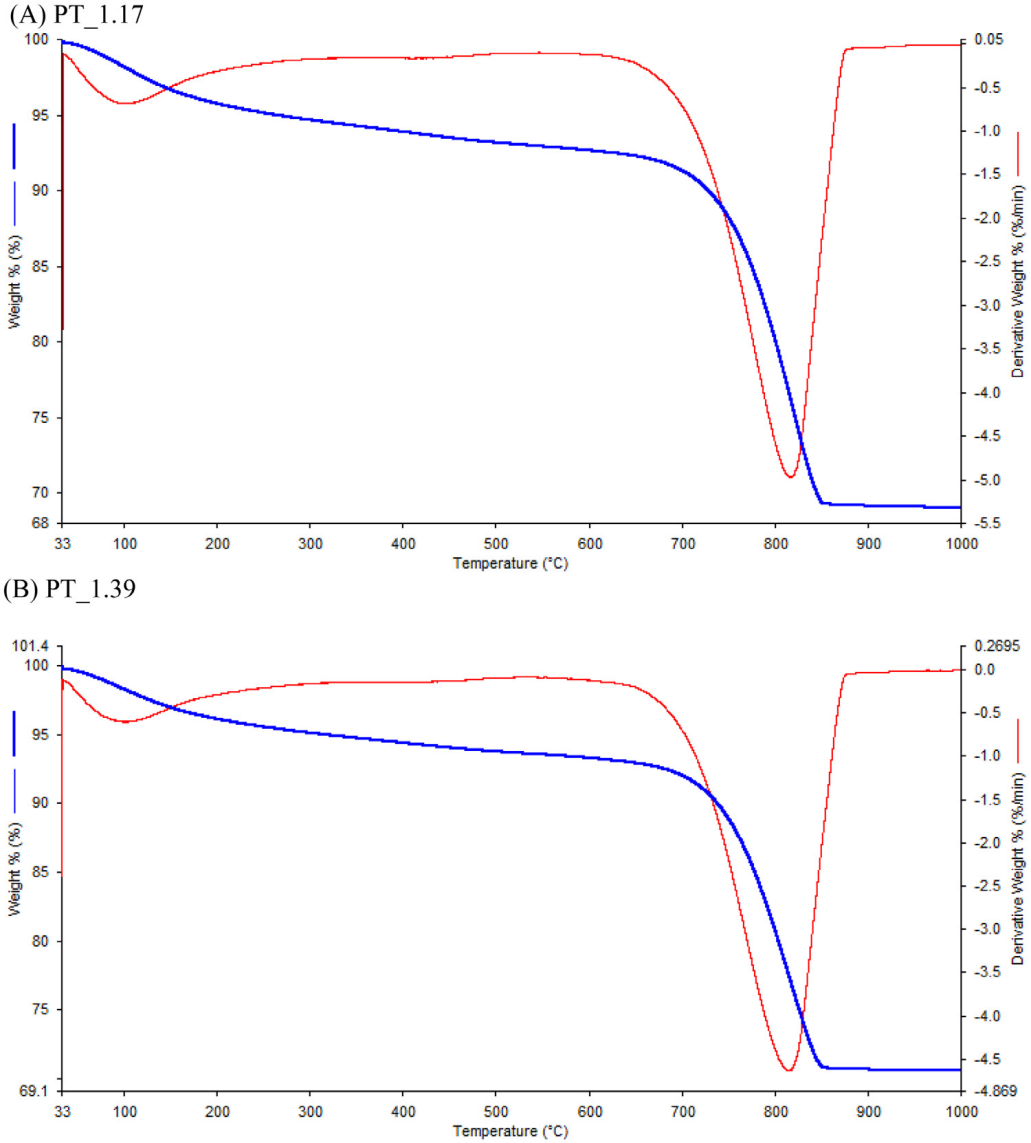
Representative TGA Representative TGA and derivative TGA (DTGA) curves of pottery sherd samples 1.17 (A), and 1.39 (B) acquired under a N_2_ atmosphere. The samples were heated at 20 °C/minute.

In this study, pottery sherds from Lake Petén Itzá, Guatemala, Central America (Fig. 1 adapted from Ref # [4]) were investigated for their composition. The data in this paper presents spectroscopic information on FTIR (Fig. 2), SEM/EDX (Figs. 3 and 4, Table 1), the approximate compositions, d-spacings and hkl patterns (Tables 2, 3, and 4). Fig. 5 depicts sample TGA/DTGA thermograms of select samples 1.12 and 1.17 showing mass losses at different temperatures in the range 33 – 1000 °C. The raw data for FTIR graphs, EDX spectra, and TGA representing Figs. 2, 4, and 5 can be found in the Supplementary Excel file titled Raw *Data FTIR* graphs, Raw EDX data graphs, and Raw data TGA graphs.

**Table 1:** Percentage elemental composition analysis (% wt/wt dry basis) of selected pottery samples using EDX.

	Percentage (%w/w) of elements in sample					
Element	1.17	1.18	1.19	1.20	1.21	1.24
C	-	-	-	-		0.7
O	47.1	49.6	46.5	38.1	46.5	49.8
Na	-	-	-	0.1	0.2	0.5
Mg	0.6	1.1	1.1	0.5	0.7	0.5
Al	10.4	3.3	3.3	7.1	10.7	12.2
Si	18.9	4.6	4.6	11.1	16.2	31.4
P	-	-	0.1		-	-
S	-	-	0.2	0.1	0.2	-
K	0.8	0.2	0.2	0.4	0.3	1.9
Ca	19.0	40.5	40.5	38.0	22.1	1.5
Ti	0.4	0.2	0.2	0.6	0.4	0.4
Fe	2.7	0.6	0.6	4.0	2.7	1.2

**Table 2:** Analysis of the Crystalline Phases, d-spacing, and h, k, l values of sample 1.01 pottery sherd from Guatemala.

Index	Angle *(2θ)*	d-Value	Net Intensity	Gross Intensity	Rel. Intensity	h, k, l	Mineral
0	6.198	14.2486	356	958	5.20%	0 0 2	Vermiculite
1	22.994	3.86476	235	355	3.40%	1 -2 -1 0, 1, 0	Gypsum Quartz
2	26.533	3.35667	65.8	173	1.00%	0 1 1 1 0 1	Quartz Quartz
						1 1 7}	Quartz
						0 1 1	
						1 0 1	
3	29.3	3.04574	1687	1785	24.60%	2 0 -2	Whewellite
4	31.345	2.85151	56	146	0.80%		Vermiculite
5	35.888	2.50005	240	319	3.50%	1 -1 -10 1 10 0 2 2 1 1 -3 -2 3 -1 -3 3 3 0 1 1 0 1 3 1 1 -1 -10	Vermiculite Alunogen Andalusite Annite Mica Laumontite Mirabilite Quartz Talc Vermiculite
6	39.374	2.2957	429	502	6.30%	1 3 3 1 -1 -10 3 -1 -8 1 1 1 0 1 2 1 0 2 1 3 6 1 -1 -11 3 1 0 1 1 -3	Chamosite 1MIIb Hexahydrite Hexahydrite Talc Talc Talc Vermiculite Vemiculite Turquoise Turquoise
7	43.145	2.809504	260	327	3.80%	0 4 2 2 1 1 3 3 2 1 3 -1 0 3 3	Gypsum Heterogenite 2H Turquoise Turquoise Turquoise
8	47.262	1.92169	121	182	1.80%	3 4 2	Tourmaline
9	47.457	1.91426	352	412	5.10%	2 6 0 3 -4 -1	Gypsum
						3 5 0}	Borax
						4 0 -6	
						6 -2 -2	
						5 -1 -5	Hexahydrite
						1 -8 -1}	Aluminite
10	48.481	1.8762	316	374	4.60%	1 5 0	
11	56.574	1.6255	81.8	137	1.20%	1 9 -3	Alunogen
						2 -2 -13}	Vermiculite
						1, 5, 6	
						1, -1, -17	
						3, -1, -7	
12	57.423	1.60345	170	226	2.50%	1 3 14	
13	60.713	1.52421	98.5	155	1.40%	3 3 2 1 5 5 0 6 0 3 -3 -1	Andalusite Borax Talc Vermiculite
14	61.398	1.5083	48.3	105	0.70%	**1 -3 1**	**Quartz**
15	63.293	1.46813	32.6	89.1	0.50%	**1 0 2** **0 1 2**	**Quartz**
16	65.896	1.41631	36	89.6	0.50%	**1 1 1**	**Quartz**
17	70.532	1.33414	26.9	82.9	0.40%	**0 2 0**	**Quartz**

**Table 3:** Analysis of the Crystalline Phases, d-spacing, and h, k, l values of pottery sample 1.02 from Guatemala

Index	Angle (2θ)	d-Value	Net Intensity	Gross Intensity	Rel. Intensity	h k l	Mineral
0	6.125	14.4181	206	839	36.20%	0 0 2	Vermiculite
1	22.944	3.87306	303	433	7.10%	0 1 0	Quartz
2	26.52	3.3583	67.6	186	6.70%	2 1 0 0 1 1 1 0 1	Aluminite Quartz Quartz
3	29.294	3.04603	4372	4481	6.10%	1 4 -1	Andalusite
4	31.331	2.85272	68.5	171	0.9%	0 1 0 1 -2 -1	Quartz Gypsum
5	35.865	2.50181	450	540	1.90%	2 -2 -2 5 1 0 5 -1 -1 6 -4 -1 1 3 2 2 2 1 3 3 0 2 -6 -1 1 -3 1 2 0 2	Brochantite Clinoptilolite Alunogen Andalusite Mirabilite Polygorskite M. Talc
6	36.10778	2.48856	157	247	2.1%	2 2 1	Andalusite
						0 1 2}	Quartz
						1 0 2	
						1 3 6	Vermiculite
						1 -2 -2}	Azurite
						2 1 0	
						1 0 4	
						3 -5 -1	Laumontite
						6 -2 -1	
						3 -3 2	PolygorskiteM
7	39.308	2.29024	738	850	6.10%	1 -2 -2 2 1 0 1 0 4	Azurite
8	43.057	2.09912	344	418	1.50%	3 -2 1	Gypsum
9	47.014	1.93126	185	253	1.60%	3 -1 1	Turquoise
9	47.393	1.9167	341	408	4.5%	3- 3 -2	Gypsum
10	48.385	1.87968	546	612	1.30%	7 -1 -3 0 0 5	Laumonite Talc
11	57.307	1.60643	296	357	3.90%	1 1 -17}	Vermiculite
						3 -1, -7	
						2 4, 6	
						0 3 3	Libethenite
12	56.467	1.6283	97.7	159	1.30%		
13	60.571	1.52744	144	206	1.9%	2 8 2 11 1 6 0 -2	actinolite
	61.2837	1.51124	75.6	137	1.0	0 6 1	Vermiculite
	61.31912	1.51041				3 3 -1	Vermiculite
	61.30665	1.50975				3 3 0	Vermiculite
14	64.74181	1.43887	161	222	0.7%	7 -1 -5	Clinoptilolite
15	69.100	1.35825	68.8	127	0.9	9 -7 -4	Clinoptilolite
16	70.155	1.34039	63	121	0.80%	-	
17	72.807	1.29797	64.7	134	0.9%	8 -2 -3 7 -1 -9	Bassanite
18	77.062	1.23656	25.2	73.8	0.3%	1 -11 -2	Gypsum

**Table 4:** Analysis of the crystalline phases, d-spacing, and h, k, l values of pottery sample 1.03.

Index	Angle *(2θ)*	d Value	Net Intensity	Gross Intensity	Rel. Intensity	h k l	Mineral
0	2.481	35.5764	12440	64561	100%	-	-
1	6.1093	14.49468	218	856	1.80%	0 0 2	Vermiculite
2	23.007	3.86253	218	366	1.80%	(0, 7, 0)	Alunogen
						(2, 2, 0)}	Bassanite
						(5, -1, -3)	
						(2, -2, -2)	
						(5, -1, -3)}	
3	29.38	3.03763	2019	2167	16.20%	(1, 1, 2)	Andalusite
4	31.421	2.8448	42.7	156	0.30%	-	-
						(2, 0, 1)	Lizardite 1M
						(3, 3, 0)}	
						(4, -2, -1)	Mirabilite
						(1, -4, -1)	
						(2, 0, -4)}	Vermiculite
						(1, -1, -10)	
						(1, 3, 2)}	
						(1, 10, 0)	Alunogen
						(2, 0, -2)	
5	35.962	2.49529	265	377	2.10%	(1, 3, 2)	
						(1, 0, 2)}	Quartz
						(0, 1, 2)	
						(1, 1, 1)	
						(1, 3, 6)	Vermiculite
						(3, -4, -1)}	
						(3, 3, -1)	Alunogen
6	39.414	2.28431	384	482	3.10%	(2, 3, 2)	Aluminite
						(5, -2, -2)}	Mirabilite
						(2, 4, 2)	
						(0, -2, 4)	Talc
7	43.181	2.09335	305	401	2.50%	(5, -1, -4)	Borax
8	47.178	1.92493	102	198	0.80%	-	
9	47.54	1.9111	263	360	2.10%	-	
						(7, -3, -2)}	Laumontite
						(1, 7, 0)	
						(0, 6, 2)	
10	48.538	1.87411	321	414	2.60%	(3, 5, 0)	Borax
11	56.634	1.62392	37	117	0.30%	(3, 4, 1) (1, 5, 1)	Libethenite
						(4, 2, 2)	Libethenite
						(1, -1, -17)}	Vermiculite
12	57.452	1.60272	124	211	1.00%	(3, -1, -7)	
						(1, 2, 1)}	Quartz
						(2, 1, 1)	
						(3, -3, 0)	Talc
						(3, -5, -6)}	Aluminite
13	60.752	1.52332	65.9	156	0.50%	(4, -5, -4)	
14	64.751	1.43855	80.5	158	0.60%	(5, 0, 2)	Diospore
15	65.727	1.41954	44	119	0.40%	(0, 2, 0)	Diospore
						(1, 0, 4)}	Quartz
						(0, 1, 4)	
						(2, 6, 1)	Talc
						(0, 2, 4)}	
						(6, 1, 0)	Andalusite
						(2, 0, 4)	
						(4, -4, -6)}	
						(11, -1, -9)	Bassanite
						(3, -13, -2)}	
						(1, -13, -2)	Clinoptilolite
16	73.032	1.29452	22.8	96.2	0.20%	(3, 3, 7)	Borax

## 2 Experimental Design, Materials and Methods

The experimental methods and procedures that allowed the data here presented are described in References # [1,[5], [6], [7] and cited references therein. Here, only the protocols for FTIR, PXRD, SEM morphological analysis, and TGA is provided, giving a large number of experimental details, usually omitted in research articles due to the words limit.

### 2.1 Study site and collection of pottery samples

The 42 samples (numbered 1.01, 1.02, 1.03, ……….1.42) that were analyzed in this study were collected from four sites of Lake Petén Itzá, Guatemala, Central America; namely, Flores, Zacpetén, Ixlú, and Nixtun Ch'ich’ (Fig. 1). The samples were tagged with their exact location and the date notating when collected (as shown in Supplementary Information Table in Ref. #1). The samples exhibited a variety of colors; tan, red, and grey (as shown in Ref # [1]) while other pieces exhibited combinations of these colors.

### 2.2 Preparation of samples and analysis

Pottery samples were crushed to a fine consistency powder using a teflon mortar and pestle.

#### 2.2.1 Fourier Transform Infrared (FTIR) analysis

An adequate concentrated layer of sample was spread on an abrasive pad and slid into the Perkin-Elmer Diffuse Reflectance Accessory. A background scan was acquired prior to acquisition of sample spectral data. In contrast to spectral data acquired via abrasive pads, energy sticks yielded absorption peaks of low intensity. A Perkin Elmer Station 100 with a CsI beam splitter scanning in the range 230 - 4000 cm^−1^ was used to acquire infrared spectra at a resolution of 4 cm^−1^. Four or more scans were run per sample. Fig. 2A - J depict FTIR spectra of all samples.

#### 2.2.2 Powder x-ray diffraction (PXRD) and scanning electron microscopy/energy dispersive X-ray spectroscopy (SEM/EDX) analysis

Powder XRD analysis was performed in the 2*θ* range of 2° - 90° on a Bruker AXS D8 Advance diffractometer equipped with an X-ray tube (Cu K_α_ radiation: λ = 1.54060 Å, 40 kV, and 40 mA) using a Ni filter equipped with a one-dimensional high-speed energy-dispersive LYNXEYE XE-T detector at scanning speed of 2 °/min and 0.0125 ° step sizes and a 1s/step.

The crystalline structure peaks were identified using the software TOPAS [3]. Samples were analyzed against 120 crystal structures in two batches of 50 each. Crystalline phases with abundance < 1.0% were removed. The pooled spectra were analyzed a second time and crystals under 1.0% were removed. After analysis of 120 crystal structures and the removal of low crystalline phases, a comprehensive list of pooled crystals was created with percentages ≥ 0.5%. Select powder XRD patterns (reported in Ref # [1]) and their *hkl* values and the crystalline phases present in pottery samples are presented in Tables 2, 3, and 4 for representative samples numbered 1.01, 1.02 and 1.03).

A Hitachi S2300 SEM was used to obtain micrographs. To minimize electrical charging, samples were sputter coated with Pd/Au. A JEOL-6100 SEM/EDX attachment and a tungsten filament was used to acquire sample composition. The detector (SiriusSD) is based on Silicon drift sensor technology and was kept at -20˚C. The working distance and voltages used were set at 15 mm, and 20 kV, respectively. An analysis period of 120 seconds was run per sample in order to lower the signal to noise ratio. Fig. 3A, 3B, 3C, and 3D depict SEM micrographs for selected samples numbered 1.13, 1.24, 1.31, and 1.35.

### 2.3 Morphological characterization of pottery samples

The pottery sherds were air dried, crushed with mortar and pestle, and analyzed with JEOL-JSM 6100 scanning electron microscope equipped with a Horiba energy dispersive X-ray spectroscopy (SEM/EDX) with an accelerating voltage of 15 kV. The surface morphology, particle diameters of samples were measured at X300, and X400 magnifications (Fig. 3A, 3B, 3C and 3D). Powder XRD patterns (previously reported in Ref. # [1], Supplementary Figure S4) and their hkl values was used to identify the crystalline structural phases present in pottery sample sherds (Tables 2, 3, and 4). Fig. 4 depicts the EDX elemental analysis of select samples numbered 1.17, 1.18, 1.19, 1.20, 1.21, 1.24, and 1.31. Except for sample # 1.31, reported in Ref. # [1], corresponding %wt/wt of elemental compositions for all other samples are shown in Table 1.

### 2.4 Thermogravimetric Analysis (TGA) analysis

Approximately 20 mg of finely crushed pottery sample was placed onto a sample holder cup. A Perkin Elmer TGA thermogravimetric simultaneous thermal analyzer (STA 6000) at 20°C/min heating rates in a nitrogen atmosphere in the range 33°C - 1000°C. Fig. 5 depicts the TGA and DTGA graphs of representative samples numbered 1.17, and 1.39.

## Declaration of Competing Interest

The authors declare that they have no known competing financial interests or personal rela- tionships which have, or could be perceived to have, influenced the work reported in this article.

## Appendix Supplementary materials

Image, application 1

Image, application 2

Image, application 3
